# Gluteus Maximus Tendon Reference: A Novel Method to Restore Leg Length in Total Hip Arthroplasty With Femoral Bone Loss

**DOI:** 10.5435/JAAOSGlobal-D-22-00149

**Published:** 2022-12-12

**Authors:** Andrew R. Ames, Amy L. Meyers, Eric T. Ballard, Michael J. Sorscher

**Affiliations:** From Ascension Genesys Hospital, Grand Blanc, MI (Dr. Meyers, Dr. Ballard, and Dr. Sorscher); Department of Orthopaedic Surgery, the Michigan State University, Statewide Campus System, East Lansing, MI (Dr. Ames, Dr. Meyers, Dr. Ballard, and Dr. Sorscher); Department of Orthopaedic Surgery, the New England Baptist Hospital, Boston, MA (Dr. Ames); and Department of Orthopaedic Surgery, the Tufts University School of Medicine, Boston, MA (Dr. Ames).

## Abstract

Accurate restoration of leg length and proximal femoral geometry is essential for achieving satisfactory outcomes in both primary and revision total hip arthroplasty. Although common landmarks such as the greater trochanter and the lesser trochanter (LT) may be used to measure limb length intraoperatively in primary cases, these landmarks may be absent or disrupted in fractures and revision procedures. We propose the distance between the gluteus maximus tendon gluteal sling (GS) and the center of the femoral head (FHC) as a novel anatomic landmark beyond the zone of injury, which can be used to restore limb length in the setting of proximal femoral bone loss or fracture.

**Methods:**

One hundred healthy hips were retrospectively reviewed using MRI to determine the distance between the proximal edge of the GS and the FHC. Additional measurements were collected including the distance between the GS and the greater trochanter and LT, as well as the FHC to the LT and greater trochanter.

**Results**: The distance between the GS and the femoral head was consistent and measured 8.0 cm (±1.88 cm, SD = 0.66). A moderate positive correlation (r = 0.37, *P* < 0.001) was observed between patient height and GS to FHC distance.

**Discussion:**

The distance between the GS and the center of the femoral head consistently measures 8 cm and can be used to set implant height to restore proximal femoral geometry and leg length in total hip arthroplasty with proximal femoral bone loss.

Accurately restoring proximal femoral geometry is essential for recreating a stable, functional total hip arthroplasty (THA) and achieving satisfactory patient outcomes.^1,2^ Numerous methods exist for intraoperative measurement of leg length discrepancy (LLD) and offset in primary total hip arthroplasties with intact proximal femoral bony architecture.^2‐4^ Although common landmarks such as the greater and lesser trochanters (LTs) can be used to measure limb length intraoperatively in primary cases, these landmarks may be absent or disrupted in fractures and revision procedures (Figure 1).^1,5^ The absence of these structures presents technical challenges to surgeons in accurately restoring biomechanics, which can result in limb length inequality, increased joint reactive forces, and altered gait kinematics, which may predispose to aseptic loosening and implant failure.^1,5‐7^ Limb length inequality is an important driver of patient-reported outcome measures and is the third most common cause of successful malpractice litigation against orthopaedic surgeons.^2,6,8‐10^

**Figure 1:**
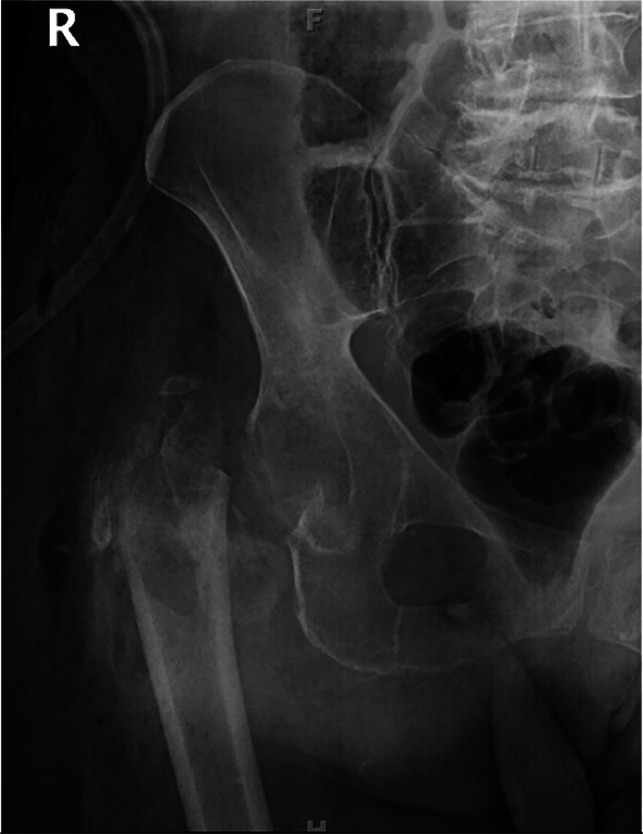
Radiograph showing examples of proximal femoral bone loss with absent bony landmarks

Various authors have described anatomic relationships in the hip and other joints to identify reference points for use in revision procedures or trauma cases requiring arthroplasty. Isik et al.^11^ evaluated several measurements on nine cadavers, including the relationship between the gluteal sling (GS) and the tip of the greater trochanter (GT) while examining methods to reduce sciatic nerve and associated arterial injuries in THA. Researchers identified the sciatic nerve 2.5 cm (±0.7 cm) from the GS and the proximal and distal insertions of the GS to the tip of the GT 7.2 cm (±0.8 cm) and 16 cm (±1.6 cm), respectively. Unnanuntana et al.^12^ examined the distances from the greater trochanter and LT to the center of the femoral head (FHC), respectively, for use in restoring leg length in THA. This cadaveric study revealed that the contralateral hip could be used to reliably determine the distance from the LT to the FHC in cases with proximal femoral bone loss but required that the LT be intact on the surgical hip as a reference point. Notably, this study confirmed data from Antapur et al., which discouraged the use of the femoral head center to GT measurement to restore limb length, finding this value demonstrated notable variability despite being commonly referenced as a reliable landmark.^1^ Thus, an accurate measurement of proximal femoral length outside of the zone of injury is needed in complex cases with proximal femoral bone loss or intertrochanteric fracture salvage to help surgeons restore ideal biomechanical relationships and leg length.

Analogous studies of proximal humeral anatomy have been conducted given the critical importance of restoring tuberosity height in proximal humerus fractures treated with arthroplasty. Murachovsky and Warner et al. demonstrated a consistent average distance from the upper edge of the pectoralis major tendon to the top of the humeral head in their cadaveric study.^13^ They concluded that this consistent distance of 5.6 cm (±0.5 cm) could be used in a fracture of the proximal humerus to determine and restore the height of the humeral head to avoid the functional consequences of tuberosity malunion.^13^ This relationship was further explored by Torrens et al.^14^ on CT scans of 20 cadavers, which demonstrated the same mean distance from the pectoralis major tendon to the level of a line tangent with the humeral head (5.64 cm), thus confirming this point of reference was reproducible and could be used to restore humeral height. Additional confirmatory studies have been conducted and demonstrated the reliability of the work of Murachovsky.^15,16^

Based on these relationships, we aimed to determine whether a consistent reference distance exists between the proximal edge of the gluteal maximus tendon GS and the FHC or the GT. Secondary measurements were also conducted to evaluate the distances between the GS and the GT and LT, as well as the FHC to the LT and GT. These values were evaluated for suitability for use as anatomic landmarks beyond the zone of injury to aid surgeons with restoring leg length and hip biomechanics in cases of complex arthroplasty with proximal femoral bone loss or fracture salvage. The data were then examined to determine whether a correlation existed between patient height and GS to FHC distance. These data were further stratified to determine whether any relationship between sex and measured distances existed.

## Methods

We conducted a retrospective chart review study of the data obtained between January 1, 2016, and January 1, 2021, at a single institution after institutional review board approval was obtained. Our study population included 50 unique patients with a total of 100 matched pair hip MRI images (female: 68, male: 32) who were evaluated from a database of all patients who underwent hip MRI (magnetic resonance imaging) at our institution. Inclusion criteria consisted of age older than 18 years, all ethnic backgrounds, both men and women, and MRI images with CPT code 72195 (MRI pelvis). Patients were excluded if they had MRI evidence of femoral head collapse or osteonecrosis, prior hip surgery, history of hip or other proximal femur fracture, and/or evidence of metastatic disease about the hip. Patient data were collected and managed using REDCap. Patient age, ethnicity, sex, and height demographics were collected for each patient.

**Figure 2:**
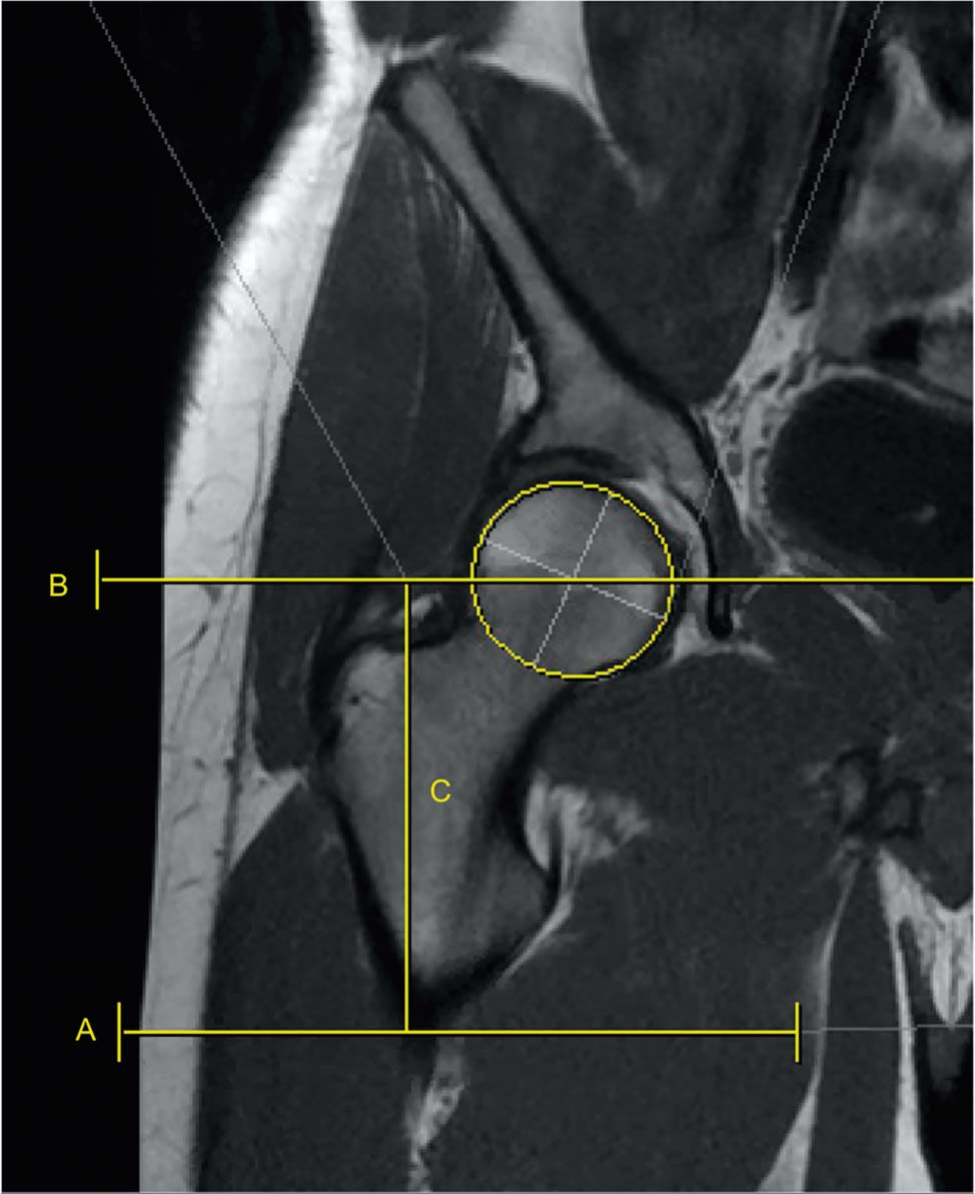
Radiograph showing line A—proximal aspect of gluteal sling (GS), line B—center of femoral head, and line C—measured distance between gluteal sling and center of femoral head.

**Figure 3:**
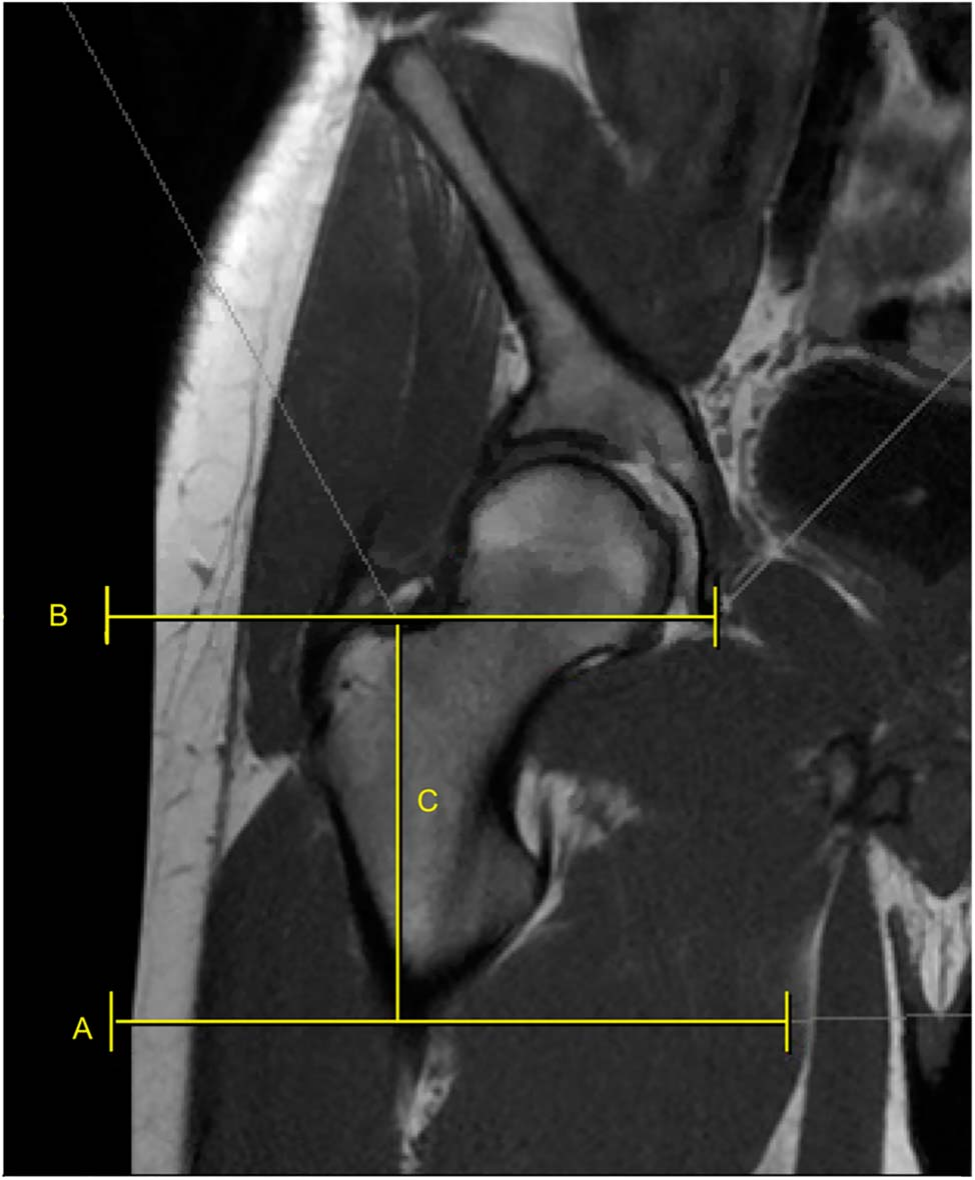
Radiograph showing line A—proximal aspect of GS, line B—tip of GT, and line C—distance between GS and tip of GT. GS = gluteal sling, GT = greater trochanter

All measurements were obtained by senior orthopaedic surgery residents using our institution's picture archive and communication system software. The GS was identified on coronal T1-weighted MRI sequences by first identifying the most proximal insertion on the gluteal tuberosity and marking it with a horizontal line. Next, a best-fit circle was drawn about the femoral head. A horizontal line was then drawn bisecting the center point indicator of the circle. The linear distance from the proximal insertion of the GS to the FHC was then measured and recorded (Figure 2). Straight line distance from the GS to the proximal aspect of the greater and LTs were also measured, respectively (Figure 3). Two additional measurements were collected from the FHC to the greater trochanter and LT, respectively.

## Data Analysis

Aggregate data for mean GS to FHC distance were calculated for all hips using descriptive statistics. This study was powered to detect a 0.5-mm difference between sexes with a 95% confidence interval (*P* < 0.05). Subgroup analysis was then conducted using a paired Student *t* test for means. Pearson correlation was used to determine whether a linear correlation existed between patient height and GS to FHC distance. Mixed-effects regression modeling was used to determine the beta value for change in measured variables by unit of height. Statistical analysis was conducted using SAS statistical software.

## Results

**Figure 4:**
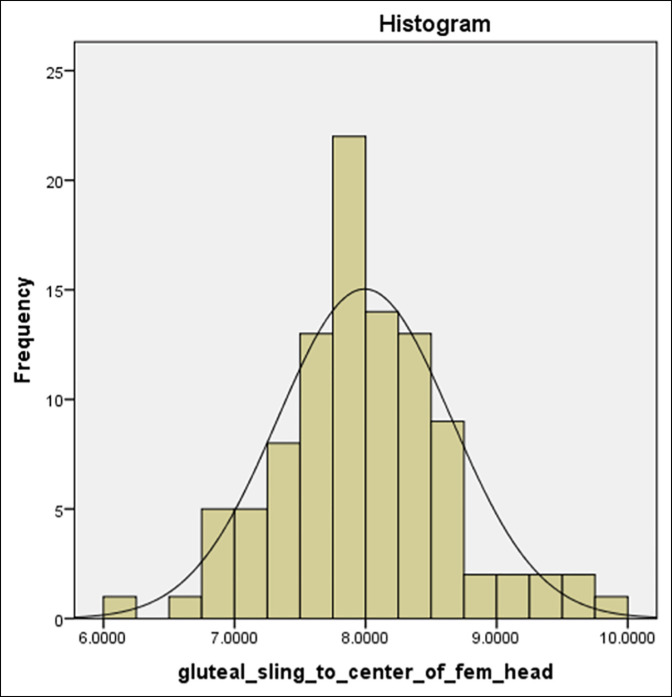
Histogram of gluteal sling (GS) to FHS distance (in centimeters).

In our study of 100 matched pair hips (female: 66, male: 34; mean age 55 years, range: 20 to 80 years), the aggregate mean distance from the GS to the FHC was 8.0 cm (range: 6.11 to 9.75 cm, SD: 0.67) (Figure 4). The mean distance from the GS to GT was 7.82 cm (range: 6.58 to 9.09 cm, SD: 0.50), and the median distance from GS to LT was 3.04 cm (range: 1.92 to 4.87 cm, SD: 0.53). The mean distance between the FHC and the GT was 0.41 cm (range: 0.0 to 1.72 cm, SD: 0.33), and the median distance from FHC to LS was 4.95 cm (range: 3.66 to 6.25, SD: 0.57). Each of the measured variables were controlled for laterality and demonstrated no significant difference between right or left hips (*P* > 0.31).

Subgroup analysis by gender revealed a statistically significant (*P* < 0.001) difference between mean GS to GT distance of 0.61 cm between male and female patients (male: 8.20 cm [SD: 0.33]; female: 7.65 cm [SD: 0.49]). Male patients demonstrated a a statistically significant (P < 0.01) 0.34 cm greater distance between GS to FHS than female patients. None of the other measurements evaluated reached significance between sexes.

**Figure 5:**
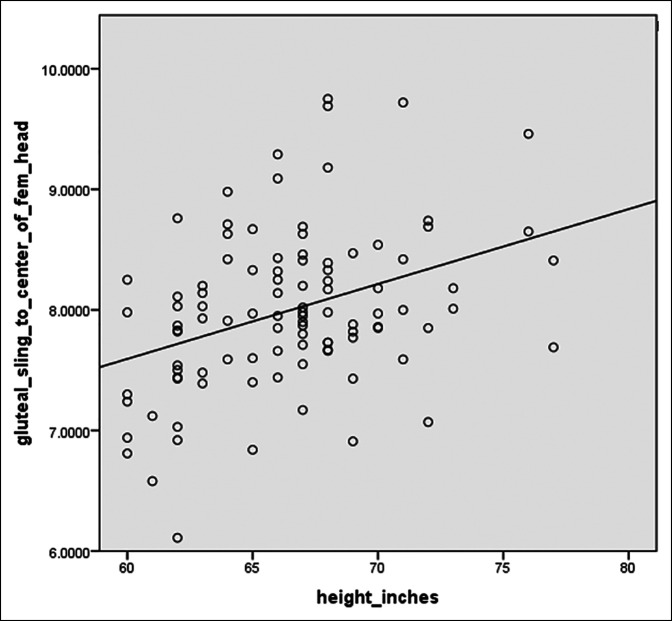
Linear regression model demonstrating increasing gluteal sling (GS) to FHS (in centimeters) distance with increasing height (in inches).

Patients had a mean height of 66.4 inches (range 60 to 77 inches). Pearson correlation linear regression modeling demonstrated a moderate strength positive correlation (r = 0.37) between height and GS to FHC distance (*P* < 0.001) (Figure 5). Mixed-effects regression modeling was then conducted and found height to be a significant predictor of GS to femoral head center distance when controlling for age and sex (*P* < 0.001). For every one inch increase in height above 66 inches, the GS to FHC distance increased by 0.91 mm per inch of height.

**Figure 6:**
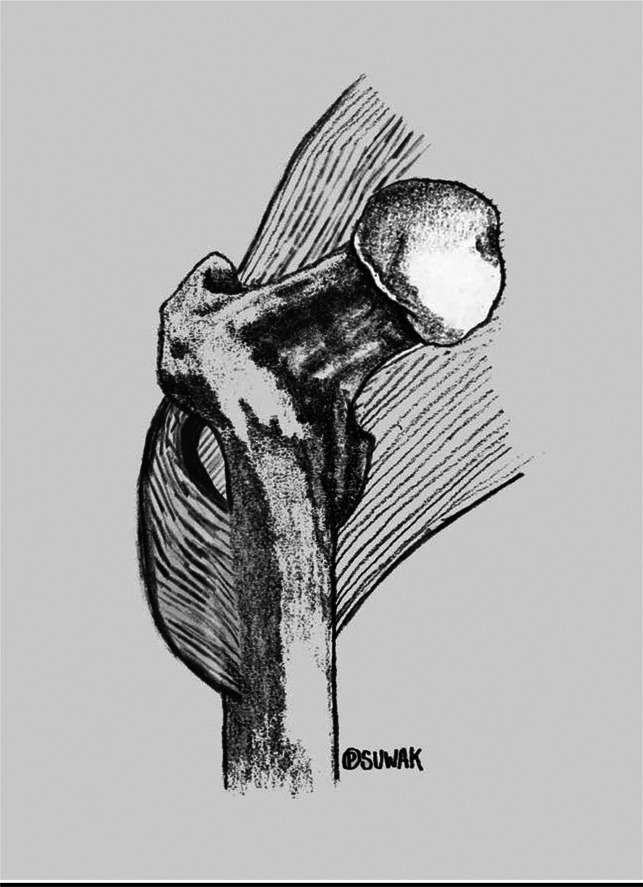
An illustration demonstrating anatomic relationship between the gluteal sling (GS), the insertion of gluteus maximus, and the proximal femur.

## Discussion

Accurately restoring leg length is difficult in complex cases of proximal femoral bone loss and presents the need for an intraoperative landmark located outside the zone of injury or degeneration. Our study demonstrated a consistent distance of 8.0 cm between the proximal edge of the GS insertion and the FHC. We propose this novel landmark and measurement in hip arthroplasty to restore hip geometry and associated biomechanical forces in cases of proximal femoral bone loss. Data analysis also demonstrated a linear relationship between height and GS to femoral head center distance. For every one inch (2.54 cm) increase in height above 66 inches (167.64 cm), the GS to FHC distance increases by 0.91 mm. Our study patients' heights ranged from 50 to 77 inches (127 to 195.58 cm), which is widely representative of most patients. While the range of GS to FHC distances in our study was within the commonly cited 2 cm of LLD, this formula can be used to tailor the leg lengths more accurately to within one millimeter based on age, sex, and height-adjusted norms.

Weber et al. found measurable and clinically notable alterations in gait kinematics occurred with greater than 10 mm of LLD and advocated for tighter tolerances than historically accepted (2 cm).^7,9^ Importantly, our study validated the prior studies by Antapur and Unnanuntana et al. discouraging the use of the tip of the GT as a reference for femoral head center.^1,12^ Our study directly measured the GT to FHC distance in 100 hips and confirmed that inadvertent leg lengthening up to 1.72 cm (range: 0 to 1.72 cm, SD: 0.61) may result from inappropriate use of this landmark.

Additional studies have identified leg length inequality as a leading cause of patient dissatisfaction after THA with high rates of associated litigation.^6^ Patterson et al.^6^ conducted a malpractice claims analysis and found LLD to be the third most common cause of litigation against orthopaedic surgeons. In this study, indemnity payments were found to approach $1 million dollars with an associated increased cost of $229,000 to insurers. These findings underscore the importance of restoring leg length and proximal femoral geometry to maximize patient outcomes.^1,7,10^

This problem is well-described in the orthopaedic literature with numerous studies conducted to identify preoperative, intraoperative, and computer-aided navigation landmarks for accurately restoring femoral length.^1,7,12^ Many of these techniques require size-matched radiographs of the contralateral hip and are dependent on limb rotation and leg position at the time of measurement. Other techniques require specialized instrumentation such as custom jigs or computer navigation.^12^ In cases of proximal femoral bone loss, such as in revision arthroplasty or arthroplasty for intertrochanteric fracture salvage, these common landmarks are absent or disrupted.^1^

GS is a familiar and easily palpable intraoperative landmark because it attaches to the gluteal tuberosity and lateral intermuscular septum. It is identifiable in all commonly used surgical approaches to the hip, and this technique requires no special equipment. Proximal femoral anatomy and leg length can be accurately restored using a ruler to set the FHC to be 8.0 cm proximal to the GS. We think that this problem is of notable interest to the orthopaedic community and that other surgeons may find this reference beneficial for restoring anatomical relationships in hip arthroplasty.

This study has several limitations. First, our institution's standard imaging protocol is to obtain 4-mm thick MRI slices, which may have contributed to potential inaccuracies in measurements for the proximal most point of the GS or trochanters. Thinner MRI slices may have improved the accuracy of measurements in our study. Isik et al.^11^ were able to grossly identify the GS on cadaveric subjects. Unlike the study by Isik et al, our study was restricted to quality of the MRI, slice sizes, and patient anatomy identifiable on representative MRI sequences. Our sample size comprised 100 hips from a homogeneous population of middle-aged Caucasian patients which may limit the generalizabilty of this study. However, our study included approximately 5 times the number of patients as similar studies used to identify the pectoralis major to humeral head distances in the shoulder.^13,14^ Our study also had an underrepresentation of male patients compared with female patients. Our study also did not control for neck-shaft angle or the relationship between leg external rotation and change in apparent neck-shaft angle and femoral head to trochanter distances.^12^ Patients with significant coxa vara or valga may have different reference distances than were found in this study; however, our study included and evaluated all comers, which may increase the generalizability of our findings. A follow-up study should be conducted to evaluate for the effect of neck-shaft angle of the hip (more varus versus valgus relationships) because this could allow for more accurate leg length reconstruction. It should also be considered that some surgeons routinely release the gluteal sling insertion which may limit the utility of this method in those instances in revision procedures. Finally, initial Institutional Review Board approval was obtained to conduct a cadaveric study; however, the coronavirus disease 2019 pandemic closed access to the cadaver specimens, and the study was converted and conducted on hip MRI images. Future studies could control for the above-mentioned variables and repeat measurements on anatomical specimens to verify our findings.

In conclusion, our study demonstrated a consistent distance of 8.0 cm between the proximal edge of the GS insertion and the FHC. We propose this novel landmark and measurement in hip arthroplasty to restore femoral geometry and associated biomechanical forces in cases with proximal femoral bone loss.
